# Krill Oil Improves Mild Knee Joint Pain: A Randomized Control Trial

**DOI:** 10.1371/journal.pone.0162769

**Published:** 2016-10-04

**Authors:** Yoshio Suzuki, Minoru Fukushima, Keishoku Sakuraba, Keisuke Sawaki, Kazuaki Sekigawa

**Affiliations:** 1 Graduate School of Health and Sports Science, Juntendo University, Inzai, Chiba, Japan; 2 Faculty of Health and Sports Science, Juntendo University, Inzai, Chiba, Japan; 3 Department of Orthopedic Surgery, School of Medicine, Juntendo University, Tokyo, Japan; 4 Fukushima Orthopedic Clinic, Nasu-Shiobara, Tochigi, Japan; University of Michigan, UNITED STATES

## Abstract

**Background:**

Krill oil is an edible oil extracted from krill, a small red-colored crustacean found in the Antarctic Ocean. The administration of krill oil is reported to mitigate inflammation in patients with cardiac disease, rheumatoid arthritis, or osteoarthritis. However, the effect of krill oil on mild knee pain has not yet been determined.

**Objective:**

To assess the effect of krill oil on mild knee pain.

**Design:**

A randomized, double-blind, parallel-group, placebo-controlled trial of fifty adults (38–85 years old) with mild knee pain attending the Fukushima Orthopedic Clinic (Tochigi, Japan) between September 2014 and March 2015.

**Interventions:**

Participants were randomized to receive 2 g per day of either krill oil or an identical placebo for 30 days.

**Outcomes:**

The primary outcome was improvement in subjective symptoms of knee pain as assessed by the Japanese Knee Osteoarthritis Measure (JKOM) and Japanese Orthopaedic Association score (JOA). Secondary outcomes included blood and urine biochemical parameters.

**Results:**

Both the placebo and krill oil groups showed significant improvements in the questions in the JKOM and JOA questionnaires after administration. After the intervention, krill oil group showed more improvements than placebo group in two questions regarding the pain and stiffness in knees in JKOM. Controlling for age, sex, weight, and smoking and drinking habits, krill oil significantly mitigated knee pain in sleeping (P < 0.001), standing (P < 0.001) and the range of motion of both right and left knees (both P = 0.011) compared to placebo. Krill oil administration raised plasma EPA (P = 0.048) and EPA/AA ratio (P = 0.003).

**Conclusion:**

This study indicates that krill oil administration (2 g/day, 30 days) improved the subjective symptoms of knee pain in adults with mild knee pain.

**Trial registration:**

UMIN-CTR; ID UMIN000014413

## Introduction

Chronic knee pain associated with aging is often considered to be an unavoidable consequence of aging, and disrupts basic activities of daily living such as walking and ascending stairs [1]. An estimated 250 million people suffer from knee osteoarthritis [2]. Commonly recommended treatments include aerobic exercise, strengthening exercises, and pharmacological agents such as acetoaminophen or non-steroidal anti-inflammatory drugs (NSAIDs) [3]. The aim of these treatments is to reduce pain, physical disability, and handi-cap [3]. However, the chronic nature of the disease has propelled researchers and patients to try various nutritional supplements to improve symptoms as well as to prevent or slow the progression of osteoarthritis [4,5].

Krill oil is an edible oil extracted from krill, a small red-colored crustacean found in the Antarctic Ocean [6]. Krill oil is rich in long-chain n-3 polyunsaturated fatty acids in the form of phospholipids [6].

Krill oil reduced tumor necrosis factor-α levels of lipopolysaccharide—stimulated peritoneal macrophages in obese Zucker rats [7]. The anti-inflammatory effect of krill oil was also observed in a rat model of dextran sulfate-induced experimental ulcerative colitis [8]. Krill oil consumption was shown to inhibit the progression of arthritis in an experimental mouse model of arthritis, in which the increase of inflammatory cytokines, IL-1 and IL-13, was suppressed [9]. Also, Deutsch et al. reported that krill oil administration mitigated the subjective symptoms of osteoarthritis as assessed by Western Ontario and McMaster Universities Osteoarthritis Index (WOMAC) and reduced C-reactive protein (CRP) levels in patients with cardiac disease, rheumatoid arthritis, or osteoarthritis with CRP levels greater than 1.0 mg/dL [10]. However, the effect of krill oil on mild knee pain has not yet been determined

The primary objective of this trial is to compare the efficacy of krill oil with a placebo in reducing the subjective symptoms of adults with mild knee pain.

## Materials and Methods

### Participants

We recruited fifty adults with mild knee pain from the Fukushima Orthopedic Clinic (Nasu-Shiobara, Tochigi, Japan) from September, 2014 to February, 2015. They had subjective symptoms of knee pain, but the pain was not severe enough to need pharmacotherapy. Exclusion criteria included candidates who were breast feeding or pregnant or would be pregnant during the study; had severe disease other than mild knee pain; were under treatment with a biological agent, such as an antibody preparation, for knee pain; and those judged inadequate to enter the trial by an orthopedist. After their eligibility was assessed by an orthopedist, candidates were invited to enroll in the trial. Candidates received detailed information about the purpose, methods, expected results, and ethical considerations (including possible adverse effects) relevant to the study. Written, informed consent was obtained from all participants.

This study was planned in cooperation with the funder and provider of the test supplements, Sunsho Pharmaceutical Co., Ltd. (Fuji, Shizuoka, Japan). This study was conducted according to the guidelines outlined in the Declaration of Helsinki. All procedures involving human subjects were approved by the Ethics Committee of Juntendo University Graduate School of Health and Sports Science (#26–13). The provider of the test supplements independently obtained study approval from their internal review board, Ethics Committee of Sunsho Pharmaceutical Co., Ltd., (#2014–6) and insured the clinical trial including product liability of the test supplements.

This study was registered at UMIN clinical trial register system (UMIN-CTR; ID UMIN000014413).

### Study design

This study was a randomized, double-blind, parallel-group, placebo-controlled trial. Participants were randomly allocated to two groups, given krill oil (2 g/d) or a placebo supplement for 30 days, and were instructed to record their daily ingestion. The compliance was calculated according to the returned diary and remaining supplements. Participants were instructed to keep their lifestyle throughout the study period.

Subjective symptoms and blood and urine parameters were determined before and after the intervention. The subjective symptoms were assessed by the Japanese Knee Osteoarthritis Measure (JKOM) [11] and Japanese Orthopaedic Association (JOA) score [12]: these were taken as primary outcome. JOA scores subjective knee pain between 0 and 100 points in total; a higher score indicates less pain; the score ranges were pain on walking; 0–30, pain on ascending or descending stairs; 0–25, range of motion; 0–35, and joint effusion; 0–10 [12]. Secondary outcomes included the blood and urine parameters. The range of motion of the knee was included in the key secondary outcomes at registration, but was excluded from the protocol because of difficulty in consistent measurement between practitioners. Blood parameters assessed were blood count, serum aspartate transaminase (AST), alanine aminotransferase (ALT), γ-glutamyl transpeptidase (γ-GTP), uric acid (UA), urea nitrogen (UN), triglyceride (TG), total cholesterol (T-Cho), HDL-cholesterol (HDL-Cho), LDL-cholesterol (LDL-Cho), C-reactive protein (CRP), matrix metalloproteinase 3 (MMP-3), and hyaluronic acid, and plasma arachidonic acid (AA), eicosapentaenoic acid (EPA), di-homo-γ-linoleic acid, and docosahexaenoic acid (DHA). Urine specific gravity and pH were also measured.

Fifty participants, 25 per group, were enrolled in the trial. The number of the participants was set according to the report by Maki et al. [13], in which the administration of krill oil (2 g/d for 4 weeks) showed significant differences in the plasma EPA & DHA concentrations of 25 participants in the control and krill oil groups [13].

Participants were randomly allocated to either the A or B group, with 25 participants per group using sealed opaque envelope containing a card printing either A or B at the enrollment. The director of the Fukushima Orthopedic Clinic allocated the group to take one envelop from the box in which the envelops were arranged in random manner at enrolment.

**Fig 1:**
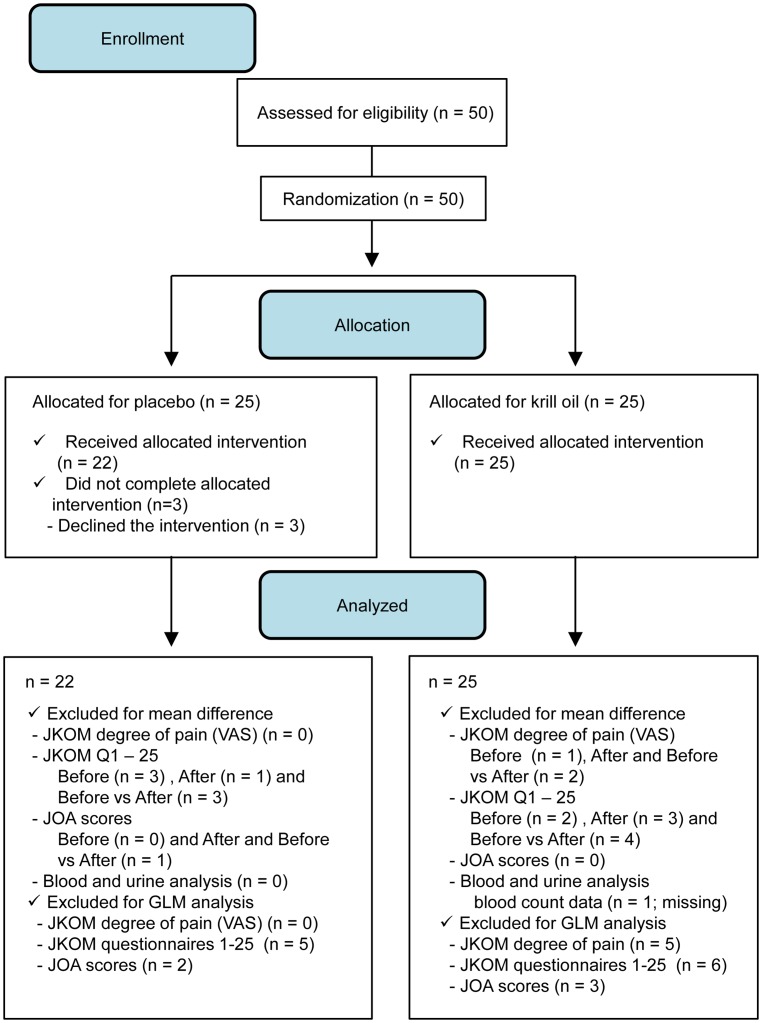
Participant flow.

There were no issues raised which required stopping the trial. Three participants, two females (aged 61 and 68 y) and one male aged 54 y, resigned from the study after enrollment due to loss of interest in completing the study. They were all belonged to the placebo group. There was no apparent difference in demographic characteristics between dropped-out and remaining participants. The remaining forty-seven participants completed the interventions and examinations. The first and the last participants were enrolled in the trial on September 22, 2014 and February 23, 2015, respectively, and were followed until October 27, 2014 and March 22, 2015, respectively. Adherence to the test administration was more than 90% in every participant; therefore, all forty-seven participants were included in the subsequent analysis (Fig 1).

After finalizing the included data, the key was opened. The participants and researchers, including orthopedists, were completely blinded from the enrollment to data finalization.

### Test supplement

Superba^™^ Krill Oil (Aker BioMarine, Lysaker, Norway) was placed in soft-capsules (250 mg oil/capsule). The placebo capsule was prepared by filling with the same amount of safflower oil (Nisshin Oillio Group, Ltd., Tokyo, Japan).

Eicosapentaenoic acid (EPA) and docosahexaenoic acid (DHA) contents in the krill oil and safflower oil for daily consumption (mg/d) were as follows: 240 mg EPA and 110 mg of DHA in krill oil, and 0 mg EPA and 0 mg of DHA in safflower oil.

The appearance of the krill oil and placebo capsules was indistinguishable. The test supplements for each participant were packed in an aluminum pouch and labelled either A or B. Participants ingested 4 capsules each at breakfast and dinner (8 capsules per day). The capsules were prepared and provided by Sunsho Pharmaceutical Co., Ltd. (Fuji, Shizuoka, Japan). The study key was kept confidential by the manufacturer until the included data was finalized. The manufacturer was not involved in the intervention, fixation, and analysis of the data.

### Biochemical analysis

White blood cell (WBC), red blood cell (RBC), hemoglobin (Hb), hematocrit (Ht), platelet (PLT), mean cell volume (MCV), mean cell hemoglobin (MCH) and mean cell hemoglobin concentration (MCHC) were analyzed using a Sysmex XE-2100 hematology analyzer (Kobe, Japan). Serum AST, ALT, γ-GTP, UA, UN, TG, T-Cho, HDL-Cho, LDL-Cho, and CRP were analyzed using a H7700 biochemistry autoanalyzer (Hitachi High-Technologies Corporation, Tokyo, Japan). Serum MMP-3 and hyaluronic acid were analyzed using a JCA-BM6050 automated analyzer (JEOL, Tokyo, Japan). The hyaluronic acid concentration of two of the participants before administration was below the detection limit (10 ng/mL), and their concentration was set as 5 ng/mL for subsequent analysis. Plasma AA, EPA, di-homo-γ-linoleic acid, and DHA were analyzed using a LC-MS 8030 system (Shimadzu, Kyoto, Japan). Urine specific gravity and pH were measured using a US-3100R automated urine analyzer (Eiken Chemical, Tokyo, Japan).

### Statistical analyses

Data were expressed as the mean ± standard deviation (s.d.) or likelihood estimated mean (LEmean) and standard error (s.e.). Mean differences between two independent groups were analyzed by a Student’s *t* test or the Mann-Whitney U test according to the normality of the data; if normality was not hypothesized even in logarithmic-transformed data, the Mann-Whitney U test was applied. Mean differences between two related groups were analyzed by a paired t test or Wilcoxon’s signed rank test according to the normality; if normality was not hypothesized even in logarithmic-transformed data, Wilcoxon’s signed rank test was applied. Comparison of numbers between two groups was analyzed by the chi-square test using Fisher’s exact probability.

In JKOM and JOA, some data were missing; the data was deleted in a listwise manner for JKOM (Q1–25) and JOA structured questionnaires. In Blood and urine analysis, blood count data was missing in one participant in krill oil group. Missing data was deleted from the analysis in a pairwise manner to compare mean difference between independent groups, and in a listwise manner to compare paired data.

The generalized linear model (GLM) was used to evaluate the effect of the supplement on changes in subjective symptoms (scores: after–(minus) before), controlling for age, sex, weight, and smoking (yes or no) and drinking (yes or no) habits. Every interaction was tested and included if it decreased Akaike’s information criteria. Participants who used anti-inflammatory analgesics were excluded. Analysis was omitted for questions that all participants had the same score either before or after the administration in either group.

Statistical analysis was performed using IBM SPSS Statistics ver. 19 (IBM Japan, Tokyo, Japan). Values of P < 0.05 were considered significant.

## Results

### Participants

Participants (22 and 25, respectively) were assigned to the placebo and krill oil groups. There were no differences between the groups in baseline characteristics, e.g., anthropometric parameters and lifestyle (Table 1). All participants complained of mild knee pain but did not require pain medication.

**Table 1:** Baselines characteristic of participants.

		Placebo		Krill oil		P	
n		22		25			
		F = 20, M = 2		F = 21, M = 4			
Age and anthropometrics							
		mean	s.d	mean	s.d	P	Method
Age	(y)	63.6	10.9	65.8	10.1	0.475	Student's t-test
Height	(cm)	156.5	7.2	155.4	7.9	0.623	Student's t-test
Weight	(kg)	58.4	9.8	57.8	11.0	0.693	Mann-Whitney U test
Lifestyle: habits and pharmacotherapy							
		n		n		P	Method
Smoking		2		6		0.470	chi-square test
Drinking		4		8		0.297	chi-square test
Anti-inflammatory analgesic		1		3		0.611	chi-square test
EPA preparation		0		1		1.000	chi-square test
Lipid-lowering drug		5		1		0.228	chi-square test

### Subjective symptoms

Both placebo and krill oil groups showed significant improvements in nearly half or more of the questions in the questionnaires after administration, except for JKOM in placebo group; improvement was significant in only 10 out of 27 items (Tables 2 & 3). Mean differences were observed in difficulty in putting socks (Q13) and heavy homework (Q18) in the JKOM scores between placebo and krill oil groups after administration (Table 2).

**Table 2:** Japanese Knee Osteoarthritis Measure (JKOM) scores before and after administration.

		Before					After					Before vs After	
		Placebo		Krill 0il		P	Placebo		Krill 0il		P	Placebo	Krill 0il
		mean	SD	mean	SD		mean	SD	mean	SD		P	P
I. Degree of knee pain													
	n	22		24			22		23			22	23
	Visual analogue scale asking the degree of knee pain. Mark an X at the level of pain during the last a few days in 10 cm horizontal line; left end (zero); "no pain at all", right end (ten); "the most severe pain you've ever had".	3.48	1.92	3.40	2.34	0.909 ^$^	2.14	2.23	1.71	2.02	0.440	0.031 *	0.003 **
*Structured questionnaire (1–25)*													
	n	19		24			21		22			18	21
II. Pain and stiffness in knees													
	[1; Not at all, 2; slight, 3; moderate, 4; quite extreme]												
1	Do you feel stiffness in your knees when you wake up in the morning?	2.21	0.85	2.33	0.82	0.593	1.67	0.80	1.41	0.73	0.171	0.013 *	0.001 **
2	Do you feel pain in your knees when you wake up in the morning?	2.16	0.76	2.13	0.80	0.914	1.71	0.78	1.36	0.49	0.122	0.008 **	0.001 **
3	How often do you wake up in the night because of pain in your knees?	1.74	1.24	1.88	0.99	0.295	1.33	0.73	1.23	0.53	0.656	0.107	0.004 **
4	Do you have pain in your knees when you walk on a flat surface?	1.84	0.69	1.96	0.75	0.672	1.48	0.81	1.36	0.49	0.977	0.021 *	0.001 **
5	Do you have pain in your knees when ascending stairs?	2.00	0.67	2.25	0.85	0.361	1.71	0.78	1.55	0.67	0.483	0.070	0.001 **
6	Do you have pain in your knees when descending stairs?	2.37	0.90	2.29	1.00	0.741	1.86	0.91	1.77	0.81	0.832	0.002 **	0.020 *
7	Do you have pain in your knees when bending to the floor or standing up?	2.42	0.90	2.46	0.83	0.568	1.81	0.75	1.82	0.73	0.945	0.012 *	0.003 **
8	Do you have pain in your knees when standing?	1.84	0.50	2.21	0.72	0.073	1.67	0.86	1.50	0.60	0.673	0.166	0.003 **
III. Condition in daily life													
	[1; Not at all, 2; a little, 3; moderately, 4; quite, 5; extremely]												
9	How difficult is ascending or descending stairs?	1.89	0.74	2.08	1.06	0.774	1.57	0.81	1.36	0.58	0.416	0.035 *	0.002 **
10	How difficult is bending to the floor or standing up?	2.42	0.84	2.21	1.06	0.375	1.76	0.94	1.64	0.85	0.668	0.006 **	0.003 **
11	How difficult is standing up from sitting on a western style toilet?	1.26	0.56	1.50	0.72	0.237	1.38	0.92	1.18	0.50	0.583	0.785	0.020 *
12	How difficult is wearing pants, skirts, and underwear?	1.47	0.70	1.42	0.65	0.793	1.38	0.74	1.19	0.51	0.283	0.257	0.279
13	How difficult is putting on socks?	1.74	0.93	1.58	0.65	0.787	1.52	0.75	1.18	0.50	0.044 *	0.132	0.013 *
14	How long can you walk on a flat surface without taking a rest?	1.47	0.61	1.33	0.64	0.312	1.29	0.56	1.14	0.47	0.218	0.157	0.083
	[1; More than 30 min, 2; about 15 min, 3; around my house, 4; can hardly walk]												
15	Have you been using a walking stick (cane) recently?	1.21	0.92	1.04	0.20	0.841	1.14	0.65	1.09	0.43	0.947	0.317	0.317
	[1; Not at all, 2; hardly, 3; sometimes, 4; often, 5; always]												
16	How difficult is shopping for daily necessities?	1.16	0.37	1.21	0.41	0.677	1.29	0.78	1.05	0.21	0.257	0.458	0.157
	[1; Not at all, 2; a little, 3; moderately, 4; quite, 5; extremely]												
17	How difficult is doing light housework (cleaning the dining room after eating, etc)?	1.32	0.58	1.21	0.41	0.617	1.24	0.77	1.05	0.21	0.492	0.480	0.317
	[1; Not at all, 2; a little, 3; moderately, 4; quite, 5; extremely]												
18	How difficult is doing heavy housework (using the vacuum cleaner, etc)?	1.68	0.89	1.33	0.48	0.212	1.52	0.93	1.09	0.29	0.047 *	0.680	0.046 *
	[1; Not at all, 2; a little, 3; moderately, 4; quite, 5; extremely]												
IV. General activities													
19	Have you gone to an event or to a department store during the last one month?	2.63	1.26	2.46	1.44	0.499	2.48	1.57	2.45	1.50	0.970	0.829	0.495
	[1; More than two to three times a week, 2; about once a week, 3; about once every two weeks, 4; once a month, not at all]												
20	Were things that you usually do (some kind of lesson, meeting friends, etc) difficult because of knee pain during the last one month?	1.68	0.89	1.33	0.56	0.170	1.33	0.80	1.14	0.35	0.557	0.273	0.083
	[1; Not at all, 2; a little, 3; moderately, 4; quite, extremely]												
21	Did you limit doing things you usually do because of knee pain during the last one month?	1.42	1.12	1.21	0.41	0.814	1.19	0.51	1.00	-	0.690	0.465	0.046 *
	[1; Not at all, 2; a little, moderately, 3; quite, 4; didn’t do them (things you do usually) at all]												
22	Did you despair of going outside somewhere close because of knee pain during the last one month?	1.42	1.02	1.08	0.28	0.208	1.14	0.48	1.00	-	0.143	0.395	0.157
	[1; Not at all, 2; hardly, sometimes, 3; often, 4; didn’t go outside (close)]												
23	Did you despair of going outside somewhere far because of knee pain during the last one month?	1.58	1.07	1.17	0.48	0.117	1.24	0.70	1.00	-	0.069	0.196	0.157
	[1; Not at all, 2; hardly, 3; sometimes, 4; often, 5; didn’t go outside (far)]												
V. Health conditions													
24	Do you think your health during the last one month is average?	2.53	0.77	2.17	0.76	0.088	2.05	0.80	2.00	0.76	0.840	0.058	0.157
	[1; Definitely yes, 2; Yes, 3; I don’t know, 4; No, 5; Not at all]												
25	Do you think that knee pain has been negatively affecting your health during the last one month?	2.16	0.90	1.79	0.83	0.159	1.24	0.77	1.00	-	0.143	0.004 **	0.002 **
	[1; Not affecting at all, 2; Affecting a little, 3; Affecting moderately, 4; Affecting significantly, 5; Affecting greatly]												
Total score (1–25)		45.63	11.75	43.63	11.06	0.659	38.00	15.06	33.50	7.82	0.223	0.016 *	0.000 **

Krill oil vs placebo; Mann-Whitney U test except for ^$^ (Student's t test),

Before vs After; Wilcoxon's signed rank test,

*; P < 0.05,

**; P < 0.01

**Table 3:** Japanese Orthopaedics Association (JOA) scores before and after administration.

		Before					After					Before vs After	
		Placebo		Krill 0il			Placebo		Krill 0il			Placebo	Krill 0il
		n = 22		n = 25			n = 21		n = 25			n = 21	n = 25
		mean	SD	mean	SD		mean	SD	mean	SD		P	P
I	Pain on walking												
	Right	28.4	2.8	28.8	2.2	0.736	29.5	1.5	29.4	1.7	0.790	0.059	0.083
	Left	28.4	2.8	29.0	2.0	0.514	29.5	1.5	29.4	1.7	0.790	0.059	0.157
II	Pain on ascending or descending stairs												
	Right	22.0	3.0	21.0	3.2	0.266	23.3	3.7	22.8	3.3	0.389	0.034 *	0.003 **
	Left	22.5	3.0	20.8	3.4	0.083	23.3	3.7	22.8	3.3	0.389	0.102	0.002 **
III	Range of motion					0.585							
	Right	31.1	3.4	31.6	3.7	0.585	32.1	3.7	33.4	2.8	0.237	0.025 *	0.007 **
	Left	31.6	3.2	31.2	3.6	0.752	32.4	3.4	33.2	2.8	0.406	0.046 *	0.004 **
IV	Joint effusion												
	Right	10.0	-	10.0	-	1.000	10.0	-	10.0	-	1.000	1.000	1.000
	Left	10.0	-	9.8	1.0	0.384	10.0	-	10.0	-	1.000	1.000	0.317
Total score													
	Right	91.6	7.6	91.4	7.7	0.939	95.0	6.9	95.6	7.0	0.642	0.001 **	0.001 **
	Left	92.5	7.4	90.8	7.7	0.429	95.2	6.6	95.4	7.1	0.750	0.006 **	0.001 **

Before vs After; Wilcoxon's signed rank test, Placebo vs krill oil; Mann-Whitney U test,

*; P < 0.05,

**; P < 0.01

In the JKOM questionnaire, all participants in krill oil group answered “Not at all” to Q21 “Did you limit doing things you usually do because of knee pain during the last one month?”, Q22 “Did you despair of going outside somewhere close because of knee pain during the last one month?”, Q23 “Did you despair of going outside somewhere far because of knee pain during the last one month?” and Q25 “Do you think that knee pain has been negatively affecting your health during the last one month? “(Table 2)

In the JOA questionnaire, none of the participants presented with edema or swelling, score 10 for IV Joint effusion in right and left knees, except for one participant in the krill oil group before administration (Table 3).

The effects of placebo and krill oil administration on changes in scores after administration were compared by GLM, controlling for age, sex, weight, and smoking and drinking habits.

The changes in JKOM scores showed that the krill oil significantly mitigated pain and stiffness in knees (Q1–8) compared to placebo (Table 4); the LEmeans were generally smaller in krill oil group and even statistically significant in questionnaires Q3 “How often do you wake up in the night because of pain in your knees?” (P < 0.001) and Q8 “Do you have pain in your knees when standing?” (P < 0.001).

**Table 4:** Changes in Japanese Knee Osteoarthritis Measure (JKOM) score.

		Placebo		Krill 0il		P
		LEmean	s.e.	LEmean	s.e.	
I. Degree of knee pain (n; placebo = 21, Krill oil = 20)		-1.04	0.76	-1.05	0.61	0.985
	Visual analogue scale asking the degree of knee pain. Mark an X at the level of pain during the last a few days in 10 cm horizontal line; left end (zero); "no pain at all", right end (ten); "the most severe pain you've ever had".					
Structured Questionnaires (n; placebo = 17, krill oil = 19)						
II. Pain and stiffness in knees						
	[1; Not at all, 2; slight, 3; moderate, 4; quite extreme]					
1	Do you feel stiffness in your knees when you wake up in the morning?	-0.78	0.32	-1.54	0.22	0.060
2	Do you feel pain in your knees when you wake up in the morning?	-1.12	0.23	-0.84	0.15	0.272
3	How often do you wake up in the night because of pain in your knees?	1.01	0.45	-1.03	0.21	0.000 **
4	Do you have pain in your knees when you walk on a flat surface?	-0.43	0.22	-0.46	0.16	0.852
5	Do you have pain in your knees when ascending stairs?	-0.44	0.24	-0.67	0.18	0.307
6	Do you have pain in your knees when descending stairs?	-0.36	0.26	-0.43	0.19	0.756
7	Do you have pain in your knees when bending to the floor or standing up?	-0.56	0.28	-0.59	0.20	0.881
8	Do you have pain in your knees when standing?	0.73	0.35	-1.03	0.14	0.000 **
III. Condition in daily life						
	[1; Not at all, 2; a little, 3; moderately, 4; quite, 5; extremely]					
9	How difficult is ascending or descending stairs?	-0.29	0.24	-0.63	0.19	0.138
10	How difficult is bending to the floor or standing up?	-1.36	0.34	-1.07	0.28	0.231
11	How difficult is standing up from sitting on a western style toilet?	0.19	0.28	-0.25	0.21	0.090
12	How difficult is wearing pants, skirts, and underwear?	-0.16	0.21	-0.41	0.16	0.214
13	How difficult is putting on socks?	-0.03	0.36	-0.53	0.19	0.226
14	How long can you walk on a flat surface without taking a rest?	-0.87	0.25	-0.15	0.12	0.006 **
	[1; More than 30 min, 2; about 15 min, 3; around my house, 4; can hardly walk]					
15	Have you been using a walking stick (cane) recently?	-0.04	0.06	0.03	0.05	0.268
	[1; Not at all, 2; hardly, 3; sometimes, 4; often, 5; always]					
16	How difficult is shopping for daily necessities?	-0.02	0.16	-0.17	0.12	0.304
	[1; Not at all, 2; a little, 3; moderately, 4; quite, 5; extremely]					
17	How difficult is doing light housework (cleaning the dining room after eating, etc)?	0.07	0.24	-0.12	0.19	0.409
	[1; Not at all, 2; a little, 3; moderately, 4; quite, 5; extremely]					
18	How difficult is doing heavy housework (using the vacuum cleaner, etc)?	-0.10	0.26	-0.14	0.20	0.840
	[1; Not at all, 2; a little, 3; moderately, 4; quite, 5; extremely]					
IV. General activities						
19	Have you gone to an event or to a department store during the last one month?	1.59	0.51	0.02	0.24	0.003 **
	[1; More than two to three times a week, 2; about once a week, 3; about once every two weeks, 4; once a month, not at all]					
20	Were things that you usually do (some kind of lesson, meeting friends, etc) difficult because of knee pain during the last one month?	-0.43	0.32	-0.16	0.25	0.376
	[1; Not at all, 2; a little, 3; moderately, 4; quite, extremely]					
V. Health conditions						
24	Do you think your health during the last one month is average?	-0.25	0.29	-0.21	0.18	0.898
	[1; Definitely yes, 2; Yes, 3; I don’t know, 4; No, 5; Not at all]					
Total score (1–25)		-10.20	3.82	-10.20	2.94	0.999

**; P < 0.01

The opposite effects were observed in questionnaires Q14 “How long can you walk on a flat surface without taking a rest?” (P = 0.006) and Q19 “Have you gone to an event or to a department store during the last one month?” (P = 0.003). For Q14, the crude score after administration showed the mean score in krill oil group was smaller than placebo group and quite close to the smallest score of 1 (Table 2), thus krill oil group should have better condition. Similarly, the crude mean score after the administration was smaller in krill oil group (Table 2), and Q19 was a question asking the outdoor experience in a previous month which did not associate the knee pain directly. Therefore, the condition drawn from this question should be almost same or better in krill oil group.

The changes in JOA scores showed the range of motion was improved by krill oil compared to placebo in both right and left knees (both P = 0.011) (Table 5).

**Table 5:** Changes in Japanese Orthopaedics Association (JOA) score.

		Placebo		Krill 0il		P
		n = 20		n = 22		
		LEmean	s.e.	LEmean	s.e.	
I	Pain on walking					
	Right	1.1	0.7	0.2	0.5	0.157
	Left	1.0	0.6	0.1	0.5	0.112
II	Pain on ascending or descending stairs					
	Right	2.4	0.9	2.4	0.7	0.972
	Left	3.0	1.0	2.9	0.6	0.917
III	Range of motion					
	Right	0.5	0.9	2.5	0.6	0.011 *
	Left	0.0	1.0	2.3	0.7	0.011 *
Total score						
	Right	4.8	1.5	5.0	1.1	0.909
	Left	6.6	3.3	9.7	2.9	0.053

*; P < 0.05

### Blood and urine parameters

Mean differences were not seen in blood count (WBC, RBC, Hb, Ht, PLT, MCV, MCH, MCHC), serum parameters (AST, ALT, γ-GTP, UA, UN, TG, T-Cho, HDL-Cho, LDL-Cho, CRP, MMP-3, and hyaluronic acid), plasma fatty acids (AA, EPA, di-homo-γ-linoleic acid, DHA and EPA/AA), and urine specific gravity and pH before administration (Table 6).

**Table 6:** Blood and urine parameters before and after administration.

		Before						After						Before vs After		
		Placebo ^&^		Krill 0il		P	Method	Placebo		Krill 0il		P	Method	Placebo	Krill 0il	
		n = 22		n = 25				n = 22		n = 25				n = 22	n = 25	
		mean	SD	mean	SD			mean	SD	mean	SD			P	P	Method
Blood cell																
White blood cell	(/μL)	5986	1545	6225	1460	0.540	2	5759	1327	5720	1352	0.950	2	0.424	0.021 *	2
Red blood cell	(x10^4^/μL)	454	32	437	35	0.103	1	455	32	435	35	0.047 *	1	0.651	0.293	1
Hemoglobin	(g/dL)	13.6	0.8	13.5	1.0	0.557	1	13.6	0.9	13.3	1.1	0.322	1	0.667	0.018 *	1
Hematocrit	(%)	42.7	2.3	42.0	2.9	0.391	1	43.0	3.0	41.8	3.1	0.199	1	0.480	0.422	1
Platelet	(x10^4^/μL)	26.0	6.3	23.8	4.7	0.224	2	25.5	5.6	23.6	4.5	0.193	1	0.329	0.411	2
MCV	(fL)	94.2	4.0	96.2	3.5	0.081	1	94.5	3.4	96.3	4.4	0.143	1	0.550	1.000	1
MCH	(pg)	30.1	1.3	30.8	1.3	0.065	1	29.9	1.4	30.6	1.3	0.085	1	0.188	0.008 **	1
MCHC	(%)	31.9	0.9	32.0	0.9	0.665	1	31.7	0.8	31.8	1.0	0.527	1	0.223	0.276	1
Serum																
Aspartate transaminase (AST)	(U/L)	20.8	4.5	22.5	6.4	0.354	2	19.9	3.4	22.2	5.7	0.113	1	0.344	0.746	2
Alanine aminotransferase (ALT)	(U/L)	18.1	7.0	18.4	7.2	0.792	2	17.0	6.9	18.3	8.2	0.495	2	0.154	0.648	2
γ-glutamyl transpeptidase (γ-GTP)	(U/L)	27.2	19.3	24.4	9.8	0.898	3	28.5	21.7	24.7	11.0	0.881	3	0.777	0.612	3
Matrix metalloproteinase 3 (MMP-3)	(ng/mL)	44.7	15.2	49.8	23.4	0.469	3	43.4	14.5	47.3	15.9	0.166	3	0.758	0.686	3
Uric acid	(mg/dL)	4.7	1.0	4.8	1.0	0.617	1	4.9	1.0	4.7	1.1	0.614	1	0.170	0.487	1
Urea Nitrogen	(mg/dL)	15.1	2.9	16.7	5.9	0.420	2	15.5	4.0	15.9	4.8	0.840	2	0.766	0.421	2
Hyaluronic acid	(ng/mL)	47.8	31.5	51.0	42.0	0.639	3	58.5	31.2	74.5	63.1	0.865	3	0.032 *	0.013 *	3
Triglyceraide (TG)	(mg/dL)	185.2	166.2	131.7	49.6	0.241	2	131.5	88.7	118.7	51.3	0.895	2	0.005 **	0.216	2
Total cholesterol (T-Cho)	(mg/dL)	209	35.0	212	37.4	0.881	3	211	32.5	212	26.5	0.915	1	0.721	0.786	3
HDL-cholesterol(HDL-Cho)	(mg/dL)	64.6	13.1	60.9	12.2	0.343	2	68.7	12.6	61.6	14.3	0.066	2	0.003 **	0.736	2
LDL-cholesterol (LDL-Cho)	(mg/dL)	111.1	27.9	123.3	34.1	0.400	3	116.9	30.3	123.5	23.2	0.399	1	0.223	0.628	3
C-reactive protein (CRP)	(mg/dL)	0.06	0.06	0.16	0.36	0.855	3	0.17	0.47	0.09	0.10	0.540	3	0.849	0.931	3
Plasma																
Arachidonic acid (AA)	(μg/mL)	218.2	49.2	218.4	57.5	0.987	1	214.7	35.0	199.3	37.7	0.155	1	0.685	0.004 **	1
Eicosapentaenoic acid (EPA)	(μg/mL)	110.5	97.3	91.0	48.1	0.823	3	90.0	79.7	115.3	48.6	0.007 **	3	0.016 *	0.048 *	3
Di-homo-γ-linoleic acid	(μg/mL)	52.9	26.5	51.1	18.2	0.920	2	50.8	19.7	45.0	17.8	0.303	2	0.925	0.005 **	2
Docosahexaenoic acid (DHA)	(μg/mL)	168.4	47.4	163.6	44.5	0.721	1	157.6	52.9	165.3	46.4	0.600	1	0.268	0.849	1
EPA/AA ratio		0.50	0.41	0.43	0.23	0.693	3	0.41	0.31	0.61	0.34	0.040 *	2	0.014 *	0.003 **	3
Urine																
Urine specific gravity		1.01	0.01	1.01	0.01	0.163	1	1.01	0.01	1.01	0.01	0.867	1	0.331	0.676	1
Urine pH		6.73	0.91	6.74	0.74	0.965	3	6.55	0.77	6.64	0.70	0.736	3	0.445	0.475	3

Placebo vs Krill oil; 1 Student's t-test、2 Log-transformed & Student's t-test, 3 Mann-Whitney U test,

Before vs After; 1 Paired t-test、2 Log-transformed & paired t-test, 3 Wilcoxon's signed rank test.

^&^ One data was missing in blood count.

*; p < 0.05,

**; P < 0.01

After administration, EPA (P = 0.048) and EPA/AA ratio (P = 0.003) were increased and AA was decreased (P = 0.004) in the krill oil group, whereas EPA and EPA/AA ratio were reduced in the control group; EPA (P = 0.016) and EPA/AA ratio (P = 0.014). Thus, EPA (P = 0.007) and EPA/AA ratio (P = 0.040) were greater in the krill oil group compared to the placebo. Also, the mean concentration of di-homo-γ-linoleic acid (an n-6 fatty acid) was significantly reduced in the krill oil group (P = 0.005) (Table 6).

After administration, RBC level was greater in the placebo group than the krill oil group (P = 0.047), whereas the change before and after administration was not significant in either group. In the krill oil group, WBC (P = 0.021), Hb (P = 0.018), and MCH (P = 0.008) were reduced significantly (Table 6), but none were outside of the reference ranges: WBC; 3300~9000/μL, Hb; Male 13.5~17.5 & Female 11.5~15.0 g/L, and MCH; 28.0~34.0 pg.

Hyaluronic acid showed an opposite change before and after administration depending on the group; the placebo group increased (P = 0.032) and the krill oil decreased (P = 0.013), but not significantly so (Table 6).

In the placebo group, TG decreased (P = 0.005) and HDL-Cho increased (P = 0.003); however, individual deviations were large and an obvious trend was not apparent (Table 6). Two participants exceeded the TG reference range of 30~149 mg/dL, with one decreasing to fall within the range. Also, only one female participant showed an increase from 82 mg/dL to 99 mg/dL, which exceeded the HDL-Cho reference range for females, 40~95 mg/dL.

The CRP level of participants was less than 1.0 mg/dL throughout the study except for two participants; one participant in the krill oil group had an initial CRP level of 1.69 mg/dL, which decreased to 0.03 mg/dL after administration, and the other participant in the placebo group had an elevated CRP level of 2.26 mg/dL after administration, from an initial level of 0.03 mg/dL.

## Discussion

Krill oil administration (2 g/d, 30 days) significantly improved mild knee joint pain assessed by JKOM and JOA compared to placebo. The plasma EPA and EPA/AA ratio increased and AA and di-homo-γ-linoleic acid decreased after administration. Hyaluronic acid increased in the placebo and decreased in the krill oil group. When changes in scores of subjective symptoms were compared by GLM controlled for age, sex, weight, and drinking and smoking habits, the improvements in krill oil group was more than those in placebo group in the pain and stiffness in knees and the range of motion of both right and left knees, significantly.

In this study, subjective symptoms of knee pain were improved in both the placebo and krill oil groups. A placebo effect could have contributed to the overall results since perception of pain is susceptible to expectancy and conditioning [14], and participants had been given an explanation of the expected effect of krill oil at enrollment. In a comparison of the effect of krill oil to that of the placebo, other possible confounding factors of knee pain were controlled for, such as age, sex, weight, smoking, drinking, and use of anti-inflammatory analgesics, and it was confirmed that krill oil significantly mitigated subjective symptoms of knee pain and range of motion of knees compared to placebo.

Previously, krill oil was reported to improve subjective knee pain in patients with systemic inflammation (CRP > 1.0 mg/dL): 300 mg/dL of krill oil reduced CRP levels in 7 days and mitigated WOMAC pain scores in 14 days to show a significant mean difference against placebo [10]. Sampalis et al. also reported that 2 g/d of krill oil administration improved knee pain by a self-assessment questionnaire for premenstrual syndrome (PMS) based on the American College of Obstetricians & Gynecologists diagnostic criteria in PMS women in 45 days; however, a significant difference was not observed compared to the control, the same amount of fish oil administration [15].

Improvements of subjective symptoms observed in this study were mainly in accordance with previous reports [10,15]. In contrast to the report by Deutcsh et al. [10], participants of the present study presented with mild knee pain but most did not have systemic inflammation (CRP > 1.0 mg/dL) throughout the study, and a significant decrease in CRP levels was not observed. Further, the participants were recruited from adults complaining of knee pain but not PMS. Therefore, improvement of knee pain should not be explained by improvement in systemic inflammation or PMS.

On the other hand, in the krill oil group, increases in plasma EPA and EPA/AA ratio were observed as expected from the report of Maki et al., in which the same effect was observed with the same amount of krill oil for 4 weeks [13]. Additionally, decreases in AA and di-homo-γ-linoleic acid were also observed. Therefore, this suggests that krill oil administration decreased the circulating n-6/n-3 ratio. As fatty acid-derived lipid mediators are involved in regulating inflammation, a decrease in the n-6/n-3 ratio is thought to suppress the production of more proinflammatory mediators [16]. In addition, krill oil contains astaxanthin and vitamins A and E: these antioxidants are known to suppress inflammation[17]. Therefore, kirll oil was suggested to mitigate subjective symptopms via anti-inflammatory activities.

Belo et al. conducted a systematic review of prognostic factors and confirmed a significant association between serum hyaluronic acid level and progression of knee osteoarthritis [18]. The kinetics of serum hyaluronic acid, observed as an increase in the placebo and a decrease in the krill oil group, could support the results of subjective symptoms: krill oil mitigated knee pain compared to placebo.

In this study, we recruited participants with mild knee pain from an orthopedic clinic located in a rural district of Japan. The lifestyle and environment of the participants, as well as their ethnic homogeneity, could affect the pathology and evaluation of subjective symptoms. The possible influence of dietary fatty acids should not be excluded since the diet was not controlled or assessed during the study; despite the instruction to keep their lifestyle was given. The subjective symptoms improved in both the krill oil and placebo groups, whereas the GLM model suggested that the krill oil improved mild knee pain better than the placebo. Improvement in knee pain is consistent with previous reports; however, the background of the participants was different. Additionally, the n-6/n-3 ratio could not be calculated for serum fatty acid composition was not measured. Therefore, in order to confirm and generalize the effect of krill oil, further study is needed.

In conclusion, this report demonstrated that krill oil could mitigate the subjective symptoms of mild knee pain. The suppression of proinflammatory lipid mediators and slowing of osteoarthritis progression were suggested by serum parameters.

## Supporting Information

S1 Table CONSORT 2010 check list. (XLSX)

S2 Table All data—containing participant, JKOM, JOA, and blood and urine analysis. (XLSX)

S1 Text Protocol. (DOCX)
