# Supplementary material for: Krill Oil Improves Mild Knee Joint Pain: A Randomized Control Trial
Source: PLoS One. 2016 Oct 4;11(10):e0162769. doi: 10.1371/journal.pone.0162769 (PMC5049853; doi:10.1371/journal.pone.0162769)
Supplement: S1 Text — (DOCX) [file pone.0162769.s003.docx]

CLINICAL PROTOCOL COVER PAGE

**Protocol Title:** A Randomized Double-Blind, Placebo-Controlled Pilot Clinical Trial to Evaluate the Effect of Krill Oil on Knee Joint Pain

**Protocol Date:** June 12,, 2014

**Study Phase:** Pilot

**Study Design:** Randomized, double-blind, placebo controlled, 2-arm parallel

**Principal Investigator** Yoshio Suzuki, Ph.D.

Graduate School of Health & Sports Science, Juntendo University

1-1, Hiraga-gakuendai, Inzai, Chiba 270-1695

**Qualified Investigator/ Orthopedist:**

Minoru Fukushima, MD, Ph.D.

1) Department of Orthopedic Surgery, School of Medicine, Juntendo University, 2-1-1, Hongo, Bunkyo-ku, Tokyo 113-8421 Japan

2) Fukushima Orthopedic Clinic, 1-10, Yoyoi-cho, Nasu-shiobara, Tochigi 325-0044 Japan

**Sponsor:** Sunsho Co. ltd.

1648 Atsuhara, Fuji, Shizuoka, 419-0201 Japan

# INTRODUCTION

Krill oil is an edible oil extracted from krill, a small red-colored crustacean found in the Antarctic Ocean [1]. Krill oil is rich in long-chain n-3 polyunsaturated fatty acids in the form of phospholipids [1].

Krill oil reduced tumor necrosis factor-α levels of lipopolysaccharide - stimulated peritoneal macrophages in obese Zucker rats [2]. The anti-inflammatory effect of krill oil was also observed in a rat model of dextran sulfate-induced experimental ulcerative colitis [3]. Krill oil consumption was shown to inhibit the progression of arthritis in an experimental mouse model of arthritis, in which the increase of inflammatory cytokines, IL-1 and IL-13, was suppressed [4]. Also, Deutsch et al. reported that krill oil administration mitigated the subjective symptoms of osteoarthritis as assessed by Western Ontario and McMaster Universities Osteoarthritis Index (WOMAC) and reduced C-reactive protein (CRP) levels in patients with cardiac disease, rheumatoid arthritis, or osteoarthritis with CRP levels greater than 1.0 mg/dL [5]. However, the effect of krill oil on mild knee pain has not yet been determined

The primary objective of this trial is to compare the efficacy of krill oil with a placebo in reducing the subjective symptoms of adults with mild knee pain.

# STUDY OBJECTIVES

To examine the effect of a 30 days (approximately 4 weeks) of krill oil administration on knee joint pain.

Primary outcomes**:**

- Japanese Knee Osteoarthritis Measure (JCOM)
- The Japanese Orthopedic Association knee rating score (JOA score)

Secondary outcomes**:**

- Blood and urine analysis
- Range of motion (ROM) of the knee

* ROM was deleted from the secondary outcome because of the difficulty to obtain the reliable data.

# STUDY DESIGN

This is a single-center, randomized, double-blind, placebo-controlled, 2-arm parallel group study. This study will consist of a 30 days administration period.

The planned sample size for this study is 50 healthy older adults, with 25 subjects randomized equally to each of the two-study arms in a double-blind manner.

| **Study Arm** | **Subject Number** |
| --- | --- |
| Krill Oil | N = 25 |
| Placebo | N = 25 |
| **Total** | **N = 50** |

In order to evaluate the goals of the study, assessments will be conducted at Visit 1 (Day 1) before administration of the test supplement (baseline), and Visit 3 (Day 31). Subject will visit or will be contacted by phone after 2 weeks of ingestion (Visit 2) in order to monitor adverse events and changes in concomitant medications as well as to foster compliance with the study dosing regimen.

The study will be conducted at a single site at Fukushima Orthopedic Clinic, Tochigi, Japan.

# SELECTION OF STUDY POPULATION

The target population for this study consists of 50 older men and women with mild knee joint pain.

Each subject will have to fulfill the inclusion criteria listed in Section 4.1. Subjects will not be included in the study if they meet any of the exclusion criteria listed in Section 4.2.

## Inclusion Criteria

1. Male or female adults with mild knee joint pain.
2. Subjects who have given voluntary, written, informed consent to participate in the study.

## Exclusion Criteria

1. Subjects who are breast feeding or pregnant or would be pregnant during the study.
2. Subjects who are under treatment with a biological agent, such as an antibody preparation, for knee pain.
3. Subjects with any major diseases of the cardiovascular, gastrointestinal, pulmonary or endocrine systems.
4. Subjects with active cancer (excluding basal cell carcinoma).
5. Subjects with neurological or significant psychiatric illnesses, including Parkinson's disease and bi-polar disorder.
6. Subjects with an allergy or sensitivity to the test supplement ingredient.
7. Subjects who are cognitively impaired and/or who are unable to give informed consent.
8. Subjects who are judged inadequate to enter the trial by Qualified Investigator.

## Concomitant Medications

Subjects who are currently taking any prescribed medications must agree to maintain their current method and dosing regimen during the course of the study.

## Early Withdrawal

**Personal reasons**

As stated in the Informed Consent Form, a subject may withdraw from the study for any reason at any time.

**Removal by Qualified Investigator:**

Subject may be discontinued at the discretion of the Qualified Investigator. A subject leaving the study prematurely will not be replaced by another. Criteria for removal of subjects from the study will include:

**Clinical reasons**

A subject may be withdrawn from the study if, in the opinion of the Qualified Investigator, it is not in the subject's best interest to continue. This includes but is not limited to adverse events related to the test supplement causing clinically significant illness, the need for prohibited concomitant medication or any participant who meets exclusion criteria during the trial. All serious adverse events will result in withdrawal from the trial. Participants who experience exacerbation of any current condition and require additional intervention or a change in their current intervention will be withdrawn from the trial.

**Protocol violation**

Any subject found to have entered this study in violation of the protocol will be discontinued from the study at the discretion of the Qualified Investigator. This will include any subject found to have been inappropriately enrolled (did not meet eligibility criteria). Subject non-compliance includes not showing up for study visits, not taking the test supplement as directed, or refusing to undergo study visit procedures.

# TEST SUPPLEMENT

## Manufacturing and Storage

The test supplement will be provided by the study sponsor Sunsho Pharmaceutical Co. Ltd. The test supplement will be carefully stored at Fukushima Orthopedic Clinic in a lockable, limited access area, accessible only to authorized study team personnel in compliance with pertinent regulations. The products will be stored at room temperature and will not be exposed to direct sunlight or heat.

All unused test supplement will be returned to the study sponsor at study closeout (within one month of last subject visit).

The test supplement is manufactured by Sunsho Pharmaceutical Co. Ltd.

## Labeling and Coding

The aluminum pouch containing the test supplement (for 30 days) will be labelled either A or B. A randomization will achieved by envelope containing a card printing either A or B at the enrollment. The sponsor keeps the key in a sealed envelope until the key open.

## Test supplements

Soft-capsule

| # | Product | Ingredient |
| --- | --- | --- |
| 1 | Placebo | safflower oil (Nisshin Oillio Group)  （250mg/capsule） |
| 2 | Krill Oil | Superba^TM^ Krill Oil  （250mg/capsule） |

## Directions

Subjects will be instructed to take 8 capsules daily, 4 capsules in the morning with breakfast and 4 capsules in the evening with dinner. Subjects will be instructed to start taking the product the day taking the baseline examination (Day 1).

## Unblinding and Allocation Concealment

Unblinding should not occur except in the case of emergency situations. In the event that a serious adverse event occurs, for which the identity of the test supplement administered is necessary to manage the subject’s condition, the treatment received by the subject will be unblinded and the test supplement identified. The sponsor must be notified and unblind the key within 24 hours.

# STUDY ASSESSMENTS

See Appendix 1 for the schedule of assessments and procedures.

## Visit 1 – Screening and enrollment

At screening, a Subject Information and Consent Form will be given to the potential subject. The subject will read the information carefully and will be given the opportunity to seek more information if needed. The subject will also be provided with the option of taking the consent form home to review prior to making his or her decision. If agreeable, the subject will sign the consent form and receive a duplicate. Once consent has been obtained, the following assessment will proceed.

- Review of the inclusion/exclusion criteria
- Review of medical history and concomitant therapies
- Collection of demographic information such as gender, age, alcohol consumption and smoking habits

Eligible subjects will be randomized and take baseline assessment.

Baseline assessments will include:

- Height, weight and BMI
- Japanese Knee Osteoarthritis Measure (JCOM)
- The Japanese Orthopedic Association knee rating score (JOA score)
- Blood and Urine analysis
- Range of motion (ROM) of the knee

Subjects will receive test supplements and administration diary.

Subjects will be instructed in detail by site personnel about the dosing regimen. The first dose of study product is to be taken the day at the dinner on the day taking the baseline assessment (Day 1).

## Visit 2 – Week 2 (Day 14 ± 3)

Subjects will return to the clinic and the following will be discussed to reinforce product compliance:

- review of current conditions, concomitant therapies and adverse events
- compliance with test supplement

The Visit 2 can be performed via phone call.

## Visit 3 – Week 8 End of Study (Day 31: 29 - 37)

Subjects will return to the clinic on day 31; Day 29– 37 will be possible according to the convenience of the subject.

Remaining test supplement in the original packaging will be returned.

Visit 3 assessments will include:

- concomitant therapies
- any adverse events
- Japanese Knee Osteoarthritis Measure (JCOM)
- The Japanese Orthopedic Association knee rating score (JOA score)
- Blood and Urine analysis
- Range of motion (ROM) of the knee
- Study diaries will be collected and reviewed.

## Clinical Assessments and Procedures

### Height, Weight and BMI

Measurement of weight and height will be performed with the subject's shoes removed, and bladder empty.

BMI will be calculated from weight and height.

### JKOM and JOA

Subjects will fill JKOM questionnaire by his/her own.

Orthopedist will fill JOA score list interviewing subject.

### Range of motion of the knee

Orthopedist will measure range of motion of the knees of subject.

### Compliance

Compliance will be assessed by counting the returned supplements at Visit 3. Compliance is calculated by determining the number of capsules taken divided by the number of capsules expected to have been taken multiplied by 100.

$$\frac{number of capsules taken}{number of capusles expected to have been taken} \times100\%$$

In the event of a discrepancy between the information in the subject diary and the amount of study capsules returned, use will be based on the capsules returned unless an explanation for loss of capsules has been provided.

If Visit 3 comes before Day 31, compliance will be calculated dividing the number of the capsules taken by the number of the capsules expected to have been taken during Day 1 to Visit 3 (8 capsules per day).

## Laboratory Analysis

Blood and urine samples will be collected at Visit 1 and Visit 3.

The following parameters will be analyzed.

### Blood count

- white blood cell (WBC)
- red blood cell (RBC)
- hemoglobin (Hb)
- hematocrit (Ht)
- platelet (PLT)
- mean cell volume (MCV)
- mean cell hemoglobin (MCH)
- mean cell hemoglobin concentration (MCHC)

### Serum parameters

- aspartate transaminase (AST)
- alanine aminotransferase (ALT)
- γ-glutamyl transpeptidase (γ-GTP)
- uric acid (UA)
- urea nitrogen (UN)
- triglyceride (TG)
- total cholesterol (T-Cho)
- HDL-cholesterol (HDL-Cho)
- LDL-cholesterol (LDL-Cho)
- C-reactive protein (CRP)
- matrix metalloproteinase 3 (MMP-3)
- hyaluronic acid

### Plasma fatty acids

- arachidonic acid (AA)
- eicosapentaenoic acid (EPA)
- di-homo-γ-linoleic acid
- docosahexaenoic acid (DHA)

### Urine

- Urine specific gravity
- pH

LSI Medience Corp (Tokyo, Japan) will be used in this study to measure laboratory parameters.

The total blood volume collected for the laboratory assessments listed above will be approximately < 20 mL at a study visit.

## Termination of the Trial

In the case of complete premature termination of the trial, participating investigators/subjects, and the Institutional Review Board must be promptly informed of the termination.

## Protocol Amendments

Alterations of the protocol may be made as the study progresses. Such changes will be captured in writing and will document the reasons for the change.

If any amendments need to be reviewed by ethical committee, it will be reviewed/approved prior to implementation.

# Safety Instructions and Guidance

As any ingredient in the test supplement is edible and generally recognized as safe, serious adverse event should not be expected to occur. However, if any adverse event will occur, it will be managed as described in this captor.

## Adverse Events and Laboratory Abnormalities

### Adverse Events

An adverse event (AE) is any untoward medical occurrence in a clinical investigation subject who has been administered a test supplement and which does not necessarily have a causal relationship with this treatment. An AE can be any unfavorable and unintended sign, symptom, or disease temporally associated with the use of a test supplement, whether or not it is considered related to that supplement. Pre-existing conditions which worsen during a study are to be reported as AEs.

During the study, subjects should record any adverse effects in their diary. At each visit the subject will be asked any difficulties or problems from the last visit. Any adverse events (AEs) will be documented and in the study record and will be classified according to the description, duration, intensity, frequency, and outcome. The investigator will assess any AEs and decide causality.

Intensity of AEs will be graded on a three-point scale (mild, moderate, severe) and reported in detail in the study record.

Mild: Awareness of event but easily tolerated

Moderate: Discomfort enough to cause some interference with usual activity

Severe: Inability to carry out usual activity

The causality relationship of test supplement to the adverse event will be assessed by the investigator as either:

Most probable: There is a reasonable relationship between the test supplement and AEs. The event responds to withdrawal of test supplement (dechallenge) and recurs with rechallenge when clinically feasible.

Probable: There is a reasonable relationship between the test supplement and AEs. The event responds to dechallenge.

Possible: There is a reasonable relationship between the test supplement and AEs. Dechallenge information is lacking or unclear.

Unlikely: There is a temporal relationship to the test supplement administration but there is no reasonable causal relationship between the test supplement and the AEs.

Not related: No temporal relationship to the test supplement administration or there is a reasonable causal relationship between non-test supplement, concurrent disease or circumstance and the AEs.

### Serious Adverse Event

A serious adverse event (SAE) is any experience that suggests a significant hazard, contraindication, side effect or precaution. It is any AE that results in any of the following outcomes:

- Death
- A life-threatening adverse event
- Hospitalization
- A persistent or significant disability or incapacity
- A congenital anomaly/birth defect in the offspring of a subject who received the study treatment
- Important medical events that may not be immediately life-threatening or result in death or hospitalization but may jeopardize the subject or may require intervention to prevent one of the outcomes listed above.

### Laboratory Test Abnormalities

The investigator must assess the clinical significance of all abnormal laboratory values as defined by the compendium of normal values for the reference laboratory.

Any treatment emergent abnormal laboratory result which is clinically significant, i.e., meeting one or more of the following conditions, should be recorded as a single diagnosis on the AEs form in the study record:

- Accompanied by clinical symptoms
- Requiring a change in concomitant therapy

This does not apply to any abnormal laboratory result which falls outside the laboratory reference range but which does not meet the clinical significance criteria or those which are a result of an AE which has already been reported.

Any laboratory result abnormality fulfilling the criteria for a serious adverse event (SAE) should be reported as such, in addition to being reported as an AE in the study record.

## Treatment and Follow-up of AEs and Laboratory Abnormalities

### Treatment and Follow-up of AEs

AEs, especially those for which the relationship to the test supplement is suspected, should be followed up until they have returned to baseline status or stabilized.

If after follow-up, return to baseline status or stabilization cannot be established, an explanation should be recorded in the study record.

### Follow-up of Laboratory Abnormalities

In the event of clinically significant unexplained abnormal laboratory test values, the tests should be repeated and followed up until they have returned to the normal range and/or an adequate explanation of the abnormality is found. If a clear explanation is established, it should be recorded in the study record.

## Reporting of SAEs and Unexpected Adverse Reactions

The Qualified Investigator will be responsible for classification of an AE as an SAE within 24 hours of notification. Causality should be signed off by the Qualified Investigator prior to reporting to ethics and regulatory bodies. Notification of any serious adverse events must be made in writing to the study sponsor. The IRB will be notified of all SAEs and unexpected adverse reactions.

# STATISTICAL EVALUATION

## Determination of sample size

Sample size is set according to the report by Maki et al. [6], in which the administration of krill oil (2 g/d for 4 weeks) showed significant differences in the plasma EPA & DHA concentrations of 25 participants in the control and krill oil groups.

**8.2 Analytical Populations**

- The **Safety Population** will consist of all subjects who received any amount of either product, and on whom any post-randomization safety information is available.
- The **Per Protocol (PP) Population** “Per Protocol (PP) Population” consists of all subjects who consumed at least 80% of test or placebo doses do not have any major protocol violations and complete all study visits. Subject who has a compliance of <80% will be deleted from the further analysis.
  1. **Analysis Plan**

An effectiveness analysis based on the per protocol population will be performed. Continuous variables will be tested for normality and log-normality. Log-normally distributed variables will be analyzed in the logarithmic domain. Non-normal variables will be analyzed by appropriate non-parametric tests.

Missing values will be deleted in a pair-wise manner for the comparison, except in the structured questionnaires of JKOM (Q1 – 25) and JOA in which missing data will be deleted in a list-wise manner.

***Statistical tests:***

Mean difference between independent groups will be analyzed by Student’s t test or Mann-Whitney U test.

Mean difference between paired groups will be analyzed by paired t test or Wilcoxon’s signed rank test.

For the primary outcomes, changes from baseline will be analyzed by generalized linear model controlling for age, sex, weight, and smoking (yes or no) and drinking (yes or no) habits. Every interaction will be tested and included if it decreases Akaike’s information criteria.

**8.3.1** **Safety**

For adverse events, a descriptive analysis will be given. Adverse events will be presented in a frequency table, by body system/group and treatment. Furthermore, nature, incidence, severity and causality will be reported for each adverse event.

# ETHICAL ASPECTS OF THE STUDY

This study will be conducted according to the guidelines outlined in the Declaration of Helsinki.

## IRB Approval

All procedures involving human subjects will be approved by the Ethics Committee of Juntendo University Graduate School of Health and Sports Science.

The provider of the test supplements will independently obtain the study approval from their IRB.

## Subject Information and Informed Consent

At screening, candidates will receive detailed information about the purpose, methods, expected results, and ethical considerations (including possible adverse effects) relevant to the study. Written, informed consent must be obtained from subject who participate this study.

## Insurance

The sponsor will insure this clinical trial including product liability of the test supplements.

# REFERENCES

1. Monograph KO. Krill oil monograph. Altern Med Rev. 2010;15: 84–86. Available: www.altmedrev.com/publications/15/1/84.pdf

2. Batetta B, Griinari M, Carta G, Murru E, Ligresti A, Cordeddu L, et al. Endocannabinoids may mediate the ability of (n-3) fatty acids to reduce ectopic fat and inflammatory mediators in obese Zucker rats. J Nutr. 2009;139: 1495–1501. doi:10.3945/jn.109.104844

3. Grimstad T, Bjørndal B, Cacabelos D, Aasprong OG, Janssen E a M, Omdal R, et al. Dietary supplementation of krill oil attenuates inflammation and oxidative stress in experimental ulcerative colitis in rats. Scand J Gastroenterol. 2012;47: 49–58. doi:10.3109/00365521.2011.634025

4. Ierna M, Kerr A, Scales H, Berge K, Griinari M. Supplementation of diet with krill oil protects against experimental rheumatoid arthritis. BMC Musculoskelet Disord. 2010;11: 136. doi:10.1186/1471-2474-11-136

5. Deutsch L. Evaluation of the effect of Neptune Krill Oil on chronic inflammation and arthritic symptoms. J Am Coll Nutr. 2007;26: 39–48. doi:10.1080/07315724.2007.10719584

6. Maki KC, Reeves MS, Farmer M, Griinari M, Berge K, Vik H, et al. Krill oil supplementation increases plasma concentrations of eicosapentaenoic and docosahexaenoic acids in overweight and obese men and women. Nutr Res. Elsevier Inc.; 2009;29: 609–615. doi:10.1016/j.nutres.2009.09.004

# APPENDICES

## Appendix 1 Schedule of Assessments

|  | **Visit 1** | **Visit 2** | **Visit 3** |
| --- | --- | --- | --- |
| DAY | **1** | **15** | **31** |
| PLACE | **clinic** | **Clinic or phone call** | **Clinic** |
| Informed Consent | X |  |  |
| Review inclusion/exclusion criteria | X |  |  |
| Review medical history | X |  |  |
| Review concomitant therapies | X | X | X |
| Randomization | X |  |  |
| Height, weight, and BMI calculated. | X |  |  |
| Range of motion of the knees | X |  |  |
| Japanese Knee Osteoarthritis Measure (JCOM) | X |  | X |
| The Japanese Orthopedic Association knee rating score (JOA score) | X |  | X |
| Laboratory Test: Blood | X |  | X |
| Laboratory Test: Urine | X |  | X |
| Test supplement Dispensed | X |  |  |
| Administration diary dispensed | X |  |  |
| Test supplement returned |  |  | X |
| Administration diary returned |  |  | X |
